# Accuracy of Urine Circulating Cathodic Antigen (CCA) Test for *Schistosoma mansoni* Diagnosis in Different Settings of Côte d'Ivoire

**DOI:** 10.1371/journal.pntd.0001384

**Published:** 2011-11-22

**Authors:** Jean T. Coulibaly, Stefanie Knopp, Nicaise A. N'Guessan, Kigbafori D. Silué, Thomas Fürst, Laurent K. Lohourignon, Jean K. Brou, Yve K. N'Gbesso, Penelope Vounatsou, Eliézer K. N'Goran, Jürg Utzinger

**Affiliations:** 1 Department of Epidemiology and Public Health, Swiss Tropical and Public Health Institute, Basel, Switzerland; 2 University of Basel, Basel, Switzerland; 3 Unité de Formation et de Recherche (UFR) Biosciences, Université de Cocody, Abidjan, Côte d'Ivoire; 4 Centre Suisse de Recherches Scientifiques en Côte d'Ivoire, Abidjan, Côte d'Ivoire; 5 Centre Hospitalier et Universitaire de Cocody, Abidjan, Côte d'Ivoire; 6 Centre de Santé Rural d'Azaguié, Departement d'Agboville, Azaguié, Côte d'Ivoire; Centers for Disease Control and Prevention, United States of America

## Abstract

**Background:**

Promising results have been reported for a urine circulating cathodic antigen (CCA) test for the diagnosis of *Schistosoma mansoni*. We assessed the accuracy of a commercially available CCA cassette test (designated CCA-A) and an experimental formulation (CCA-B) for *S. mansoni* diagnosis.

**Methodology:**

We conducted a cross-sectional survey in three settings of Côte d'Ivoire: settings A and B are endemic for *S. mansoni*, whereas *S. haematobium* co-exists in setting C. Overall, 446 children, aged 8–12 years, submitted multiple stool and urine samples. For *S. mansoni* diagnosis, stool samples were examined with triplicate Kato-Katz, whereas urine samples were tested with CCA-A. The first stool and urine samples were additionally subjected to an ether-concentration technique and CCA-B, respectively. Urine samples were examined for *S. haematobium* using a filtration method, and for microhematuria using Hemastix dipsticks.

**Principal Findings:**

Considering nine Kato-Katz as diagnostic ‘gold’ standard, the prevalence of *S. mansoni* in setting A, B and C was 32.9%, 53.1% and 91.8%, respectively. The sensitivity of triplicate Kato-Katz from the first stool and a single CCA-A test was 47.9% and 56.3% (setting A), 73.9% and 69.6% (setting B), and 94.2% and 89.6% (setting C). The respective sensitivity of a single CCA-B was 10.4%, 29.9% and 75.0%. The ether-concentration technique showed a low sensitivity for *S. mansoni* diagnosis (8.3–41.0%). The specificity of CCA-A was moderate (76.9–84.2%); CCA-B was high (96.7–100%). The likelihood of a CCA-A color reaction increased with higher *S. mansoni* fecal egg counts (odds ratio: 1.07, p<0.001). A concurrent *S. haematobium* infection or the presence of microhematuria did not influence the CCA-A test results for *S. mansoni* diagnosis.

**Conclusion/Significance:**

CCA-A showed similar sensitivity than triplicate Kato-Katz for *S. mansoni* diagnosis with no cross-reactivity to *S. haematobium* and microhematuria. The low sensitivity of CCA-B in our study area precludes its use for *S. mansoni* diagnosis.

## Author Summary

We aimed to assess the accuracy of a commercially available rapid diagnostic test for the detection of an infection with the blood fluke *Schistosoma mansoni* in urine. In total, 446 school children from three different settings of south Côte d'Ivoire provided three stool and three urine samples. Stool samples were examined with the widely used Kato-Katz technique and analyzed with a microscope for *S. mansoni* eggs. Urine samples were examined with a filtration method for *S. haematobium* eggs and with a rapid diagnostic test for *S. mansoni* that is based on detecting circulating cathodic antigens (CCA). We used a commercially available test (designated CCA-A) and an experimental formulation (CCA-B). Examination of nine Kato-Katz thick smears per child revealed a prevalence of S. *mansoni* in the three settings of 32.9%, 53.1%, and 91.8%. The sensitivity of triplicate Kato-Katz from the first stool sample was comparable to a single CCA-A (47.9–94.2% *vs.* 56.3–89.6%), and significantly higher than the sensitivity of a single CCA-B test (10.4–75.0%). CCA-A showed a considerably lower specificity than CCA-B (76.9–84.2% *vs.* 96.7–100%). In the settings studied in south Côte d'Ivoire, the CCA-A test holds promise for the diagnosis of *S. mansoni*, whereas results with CCA-B were suboptimal.

## Introduction

There is growing awareness, political commitment, and financial resources to control neglected tropical diseases (NTDs) [1]–[3]. Preventive chemotherapy, that is the repeated large-scale administration of drugs to at-risk populations, has become the key strategy for the control of several NTDs, including schistosomiasis [3]–[5]. Although the issue of diagnosis has received only token attention in the current era of preventive chemotherapy, its importance must be emphasized for rapid identification of high-risk communities warranting regular treatment, appraisal of drug efficacy, monitoring progress of control interventions, and improved patient management [6]–[8]. With regard to intestinal schistosomiasis due to *Schistosoma mansoni* and *S. japonicum*, the Kato-Katz technique is the most widely used diagnostic approach in epidemiological surveys [8], [9]. Although the Kato-Katz technique is relatively simple to perform, it requires a minimum of equipment (i.e., microscope, chemicals, and test kit material) and well-trained laboratory technicians [10]. Moreover, a shortcoming of the Kato-Katz technique is the only low-to-moderate sensitivity for *S. mansoni* diagnosis in low endemicity areas [11]–[13]. Hence, multiple Kato-Katz thick smears are required to enhance sensitivity [14], but this poses operational challenges and strains financial resources.

The detection of circulating antigen of *S. mansoni* in urine has been suggested as an alternative to the Kato-Katz technique [15]–[17]. Indeed, both circulating anodic antigen (CAA) and circulating cathodic antigen (CCA) can be detected in sera and urine of individuals infected with *S. mansoni* [18]. Both antigen-detecting assays are sensitive and specific and correlate with the presence and intensity of infection [19]. Antigen detection in urine using a rapid diagnostic test (RDT) based on an enzyme-linked immunosorbent assay (ELISA) technique is potentially useful and non-invasive and could change the management of infected individuals, particularly at the peripheral level in endemic countries where microscopes and qualified laboratory technicians are often not available [6], [7]. A point-of-contact (POC) CCA urine test has been developed for the diagnosis of *S. mansoni* [15], which is now commercially available as a RDT in cassette form. In view of promising results obtained thus far [17], [20], [21], the Schistosomiasis Consortium for Operational Research and Evaluation (SCORE) initiated a multi-country study to assess the accuracy of a commercially available CCA cassette test for the diagnosis of *S. mansoni*.

The study reported here is part of this multi-country evaluation. We assessed the accuracy of a commercially available urine CCA cassette test (designated CCA-A) for *S. mansoni* diagnosis. Additionally, we employed an experimental formulation of the test (CCA-B). Nine Kato-Katz thick smears from each participant served as diagnostic ‘gold’ standard. In addition, our team employed the ether-concentration method on sodium acetate-acetic acid-formalin (SAF)-fixed stool samples for the diagnosis of *S. mansoni*, urine filtration for the identification of *S. haematobium* eggs, and Hemastix dipsticks for the detection of microhematuria in urine. The study was carried out in south Côte d'Ivoire, in three settings where *S. mansoni* is endemic at different levels, whereas *S. haematobium* co-exists in one of the settings.

## Methods

### Ethics Statement

The study protocol was approved by the institutional research commission of the Swiss Tropical and Public Health Institute (Basel, Switzerland) and was cleared by the ethics committees of Basel (EKBB; reference no. 377/09) and Côte d'Ivoire (reference no. 1993 MSHP/CNER). District health and education authorities, village chiefs, parents/legal guardians, and participating children were informed about the purpose and procedures of the study. Parents/legal guardians provided written informed consent for their children to participate. Additionally, all children assented orally. Participation was voluntary and children could withdraw at any time without further obligation. All parasitological results were coded and treated confidentially. At the end of the study, children attending the schools involved in this study were treated with praziquantel (single 40 mg/kg oral dose) and albendazole (single 400 mg oral dose) free of charge, irrespective of the child's helminth infection status [22].

### Study Area and Population

In October/November 2010, we carried out a cross-sectional survey in three epidemiological settings in the district of Azaguié, south Côte d'Ivoire. Azaguié is located approximately 40 km north of Abidjan, the economic capital of Côte d'Ivoire. The settings were selected after a pre-screening done in 10 schools. For the pre-screening, in each school, 25 children were randomly selected. All children attending grades 3–5 (CE1, CE2, and CM1) were given a unique number, lots including all numbers were closed and placed in a box, and finally 25 lots per school were drawn. The selected children provided a single stool and a single urine sample, which were examined for *S. mansoni* with triplicate Kato-Katz thick smears and *S. haematobium* with a single filtration, respectively. Based on this pre-screening, we selected the following sites, according to SCORE guidelines: setting A, low *S. mansoni* endemicity (i.e., prevalence: 10–24%); setting B, moderate *S. mansoni* endemicity (prevalence: 25–49%); and setting C, co-endemic for *S. mansoni* and *S. haematobium*.

### Sample Size

According to the literature, a single Kato-Katz thick smear for diagnosis of *S. mansoni* in low endemicity settings has a sensitivity of only 20–30% [23], [24]. However, since our study was to be carried out in both low and moderate endemicity settings, we assumed that a single Kato-Katz thick smear has a maximum sensitivity of 60%. The sensitivity of the CCA test is reported to be 80% or higher [15], [25]. Using these sensitivity estimates, a significance level of 5%, and a power of 80%, our sample size of complying children was calculated at 90. Assuming a compliance of 70% for the submission of each of three requested stool samples, the number of children to be included in each study setting was at least 199. To achieve this sample size, we selected by computer-based randomization 220 children aged 8–12 years from readily available school lists of Abbé-Begnini (setting A), Azaguié Gare (setting B), and M'Bromé/Makouguié (setting C).

### Field Procedures

The purpose and procedures of the study were explained to the village authorities, the school directors, and the teachers of the selected schools. Teachers were invited to prepare class lists, including names, sex, and age of the children attending grades 3–5. Next, the study was explained to the children in lay terms and they were provided with an information and consent sheet with further details of the study and children and parents' rights. Children who submitted a written informed consent from their parents/guardians and assented orally themselves were given a 125 ml plastic container labeled with a unique identifier (ID). Children were invited to return the containers filled with a fresh lime-sized morning stool sample the following day. Upon collection of the filled container, a new empty container was handed out for stool collection on the next day. This procedure was repeated over a week until most children had submitted a total of three stool samples. Each day, between 10:00 and 12:00 hours, participating children were provided with another empty container labeled with the respective ID for collection of urine samples.

### Laboratory Procedures

Stool and urine samples were transferred to a laboratory at the Université de Cocody and processed the same day. From each stool sample, triplicate Kato-Katz thick smears were prepared, using 41.7 mg templates, following standard protocols [9]. In brief, triplicate Kato-Katz thick smears were prepared on microscope slides, labeled with a child's ID plus letter A, B, or C. Slides were allowed to clear for at least 30 min before quantitative examination under a microscope by experienced laboratory technicians. The number of *S. mansoni* and other helminth eggs (e.g., *Ascaris lumbricoides*, hookworm, and *Trichuris trichiura*) was counted and recorded for each species separately. For quality control, 10% of the Kato-Katz thick smears were re-examined by a senior technician.

In addition, from the second day stool sample, ∼1 g of feces was weighed into plastic vials containing 10 ml of a SAF solution. Within 8 weeks, the SAF-fixed stool samples were processed with the ether-concentration method, following a standard protocol [12], [26]. In brief, the stool-SAF solution was rigorously shaken and then poured through medical gauze placed on a plastic funnel into a conical glass tube. The conical tubes were centrifuged for 1 min at 500× *g*. Subsequently, the supernatant was discarded and 7 ml of 0.85% sodium chloride (NaCl) solution and 2–3 ml ether were added to the pellet. Tubes were closed with a rubber stopper, manually shaken for ∼30 sec and then centrifuged for 5 min at 500× *g*. This procedure leads to the separation of the suspension in four layers. The three top layers were discarded and the complete sediment layer was placed on a microscope slide, covered with a slip and subsequently examined under a microscope for helminth eggs (i.e., *S. mansoni* and soil-transmitted helminths) and intestinal protozoon cysts.

All urine samples were subjected to CCA-A (batch 32727) on the day of sample collection. The first urine sample was additionally subjected to CCA-B (batch 32686). Both CCA urine cassette assays were obtained from Rapid Medical Diagnostics (Pretoria, South Africa) and performed at ambient temperature, following the manufacturer's instructions. Briefly, one drop of urine was added to the well of the testing cassette and allowed to absorb. Once fully absorbed, one drop of buffer (provided with the CCA test kits) was added. The test results were read 20 min after adding the buffer. In case the control bands did not develop, the test was considered as invalid. Valid tests were scored as either negative or positive, the latter further stratified into 1+, 2+, or 3+ according to the visibility of the color reaction. All tests were read independently by two blinded investigators and in case of discordant results discussed with a third independent investigator until agreement was reached.

In addition to the CCA cassettes, each urine sample was subjected to a filtration method for *S. haematobium* egg counts and to a Hemastix dipstick (Siemens Healthcare Diagnostics GmbH; Eschborn, Germany) for microhematuria assessment on the day of sample collection. In brief, samples were shaken, and 10 ml of urine filtered through a 13-mm diameter small meshed filter (20 µm; Sefar AG; Heiden, Switzerland), which was then placed on a labeled slide and examined under a microscope for *S. haematobium* eggs [8]. For appraisal of microhematuria, a Hemastix dipstick was soaked in urine, left in the open air for 1 min, before scoring according to the manufacturer's instructions.

### Statistical Analysis

Data were entered twice in a Microsoft Excel spreadsheet, transferred in EpiInfo version 6.4 (Centers for Disease Control and Prevention; Atlanta, GA, USA) and validated. Statistical analyses were done with STATA version 10 (Stata Corp.; College Station, TX, USA).

Only those children who had complete data records were included in the final analysis (i.e., nine Kato-Katz thick smears, a single ether-concentration, three CCA-A, one CCA-B, three urine filtrations, and three Hemastix dipsticks). To obtain a standardized measure of infection intensity, expressed as eggs per gram of stool (EPG), for each individual, we calculated the arithmetic mean *S. mansoni* fecal egg counts (FECs) from the nine Kato-Katz thick smears and multiplied by a factor 24. Infection intensity of *S. mansoni* was classified into light (1–99 EPG), moderate (100–399 EPG), and heavy (≥400 EPG). Egg counts of *S. haematobium* were utilized to stratify into light (1–49 eggs/10 ml of urine) and heavy infection intensities (≥50 eggs/10 ml of urine) [4].

The strength of agreement between nine Kato-Katz thick smears and triplicate CCA-A, one CCA-B, and one ether-concentration for each endemicity setting was assessed by kappa statistics (κ), as follows: κ<0 indicating no agreement, κ = 0–0.2 indicating poor agreement, κ = 0.21–0.4 indicating fair agreement, κ = 0.41–0.6 indicating moderate agreement, κ = 0.61–0.8 indicating substantial agreement, and κ = 0.81–1.0 indicating almost perfect agreement [27], [28].

As proposed by the SCORE secretariat, the results from nine Kato-Katz thick smears were considered our ‘gold’ standard. We determined the sensitivity (proportion of true-positives detected by the test) and specificity (proportion of true-negatives detected by the test) of single and multiple tests. As with some of our previous work, we used a second ‘gold’ standard by considering a positive test result (regardless of the test) as true-positive [29], [30]. Hence, we combined results from all tests (i.e., nine Kato-Katz thick smears plus triplicate CCA-A, one CCA-B, and one ether-concentration) and therefore maximized specificity.

We employed an ordinal logistic regression approach, which is an extension of the general linear model to ordinal categorical outcomes to assess the correlation between CCA-A and CCA-B color reaction categories and *S. mansoni* FECs. The arithmetic mean FEC of three Kato-Katz thick smears per stool sample per day served as continuous explanatory variable, whereas the color reaction of the CCA test was considered as categorical outcome. This statistical procedure was also used to compare between the CCA test results considered as categorical outcome, and different infection intensity categories of *S. mansoni* (i.e., light, moderate, and heavy) utilized as categorical explanatory variables.

A logistic regression was performed to assess the association between CCA-A and CCA-B test results, expressed as binary outcome variable (negative/positive) with *S. haematobium* egg count as continuous explanatory variable and mircohematuria as categorical explanatory variable among children without a *S. mansoni* infection.

Non-overlapping 95% confidence intervals (CI) or p-values≤0.05 were considered as statistical significance.

## Results

### Study Adherence

**Figure 1:**
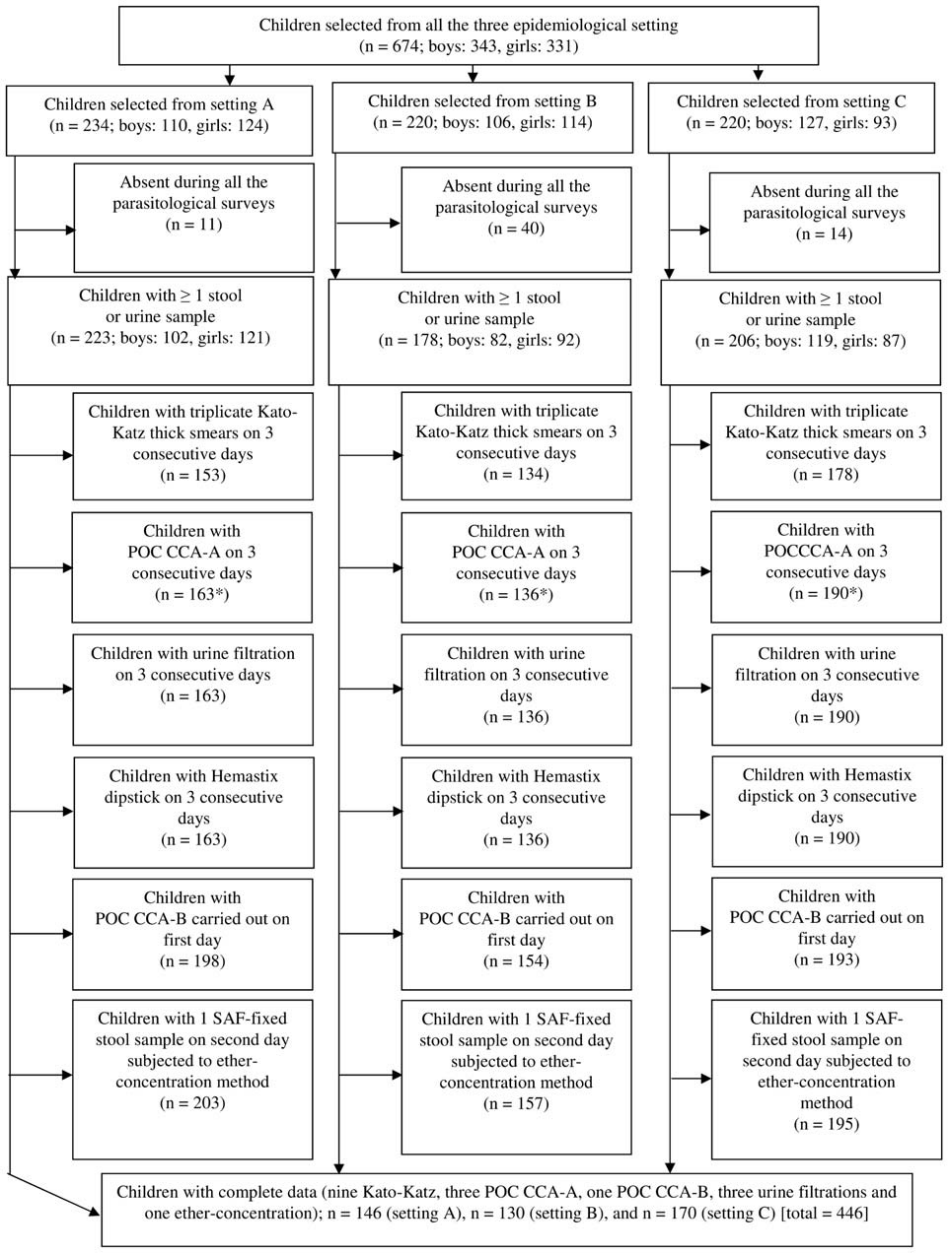
STARD-flowchart showing study participation, stratified by epidemiological setting. Flowchart detailing the study participation and adherence of children for multiple stool and urine submissions for diagnosis of *S. mansoni* and *S. haematobium* infections in Azaguié, south Côte d'Ivoire, in October and November 2010. According to nine Kato-Katz thick smear examinations, the prevalence of *S. mansoni* in setting A, B and C was 32.9%, 53.1% and 91.8%, respectively. Of note, *S. haematobium* is co-endemic in setting C.

Figure 1 shows the adherence of school children to provide multiple stool and urine samples for a suite of diagnostic tests for detection of *S. mansoni* and *S. haematobium* infection. Overall, 674 school children aged 8–12 years were enrolled with slightly more boys than girls (343 *vs.* 331). The number of children in settings A, B and C was 234, 220 and 220, respectively. At least one stool or one urine sample was provided by 223, 178 and 206 children in settings A, B and C, respectively. Overall, 465 children submitted three stool samples, which were subjected to triplicate Kato-Katz thick smears. Results from a single ether-concentration method were available for 555 children. Three CCA-A test results were available for 489 children, whereas 545 children had the first urine sample additionally subjected to a CCA-B test. Finally, three urine filtrations for *S. haematobium* diagnosis and three Hemastix dipstick tests for appraisal of microhematuria were done for 489 children.

Results on three stool samples (examined with nine Kato-Katz thick smears and a single ether-concentration) and three urine samples (examined with three CCA-A, one CCA-B, three urine filtrations and three Hemastix dipsticks) were available from a total of 446 children. Among them 48.7% (n = 217) were boys and the median age of the cohort was 10 years. All further analysis focused on this cohort of children.

### *S. mansoni* and *S. haematobium* Infection

Table 1 shows the number of children examined and those positive for *S. mansoni* and *S. haematobium*, as assessed by different diagnostic approaches, stratified by study setting.

**Table 1:** Prevalence of *S. mansoni* and *S. haematobium* according to different diagnostic approaches, stratified by epidemiological setting.

Diagnostic approach	Setting A			Setting B			Setting C		
	No. of children tested	No. of children positive	% positive (CI*)	No. of children tested	No. of children positive	% positive (CI*)	No. of children tested	No. of children positive	% positive (CI*)
*S. mansoni* diagnosis									
Nine Kato-Katz thick smears	146	48	32.9 (25.2–40.6)	130	69	53.1 (44.4–61.8)	170	156	91.8 (87.6–95.9)
Three CCA-A**	146	49	33.8 (26.0–41.6)	130	60	48.8 (39.8–57.7)	170	141	85.9 (80.6–91.3)
One CCA-B*	146	5	3.4 (0.3–4.7)	130	22	17.2 (10.6–23.8)	170	117	68.8 (61.8–75.9)
One ether-concentration	146	6	4.1 (0.8–7.4)	130	9	6.9 (2.5–11.3)	170	65	38.2 (30.8–45.6)
*S. haematobium* diagnosis									
Three Hemastix dipsticks (excluding trace results)	113	6	5.3 (1.0–9.5)	86	5	6.7 (1.4–12.1)	92	41	44.6 (32.2–54.9)
Three urine filtrations	146	6	4.1 (0.8–7.4)	130	1	0.8 (0.0–2.2)	170	111	65.3 (58.1–72.1)

The study was carried out in three epidemiological settings of south Côte d'Ivoire in October and November 2010. Triplicate Kato-Katz thick smears from the first collected stool sample, nine Kato-Katz thick smears from three stool samples, one CCA-A from the first collected urine sample, three CCA-A tests from three urine samples, one CCA-B test from the first collected urine sample, and one ether-concentration test on SAF-fixed stool samples from the second collected stool sample were used for the diagnosis of *S. mansoni*. Three urine filtrations were employed for *S. haematobium* diagnosis and three Hemastix dipsticks were used for microhematuria appraisal.

*Exact 95% confidence interval.

**In settings A, B and C, there were 2, 6 and 7 tests considered invalid, and hence not taken into account for prevalence calculations.

#### Kato-Katz technique and ether-concentration method

In setting A, B, and C, according to nine Kato-Katz thick smears examined per child, the observed prevalence of *S. mansoni* was 32.9%, 53.1% and 91.8%, respectively. In settings A and B, most of the infections were of light intensity (96.2% and 68.1%, respectively), whereas in setting C, three-quarter of the children had moderate or heavy infection intensities (76.2%). The lowest arithmetic mean FEC was found in setting A (17.4 EPG, 95% CI: 0–38.9 EPG) and the highest arithmetic mean FEC in setting C (482.8 EPG, 95% CI: 388.1–577.4 EPG). In setting B, the arithmetic mean FEC was 62.4 EPG (95% CI: 36.2–88.5 EPG).

Considerably lower *S. mansoni* prevalence estimates were obtained after subjecting a single stool sample to an ether-concentration method, 4.1%, 6.9% and 38.2% in setting A, B and C, respectively.

#### CCA test results

In setting A, B, and C, the respective prevalence of *S. mansoni* based on triplicate CCA-A tests were 33.8%, 48.8%, and 85.9%. Out of the 570 CCA-A tests performed on three consecutive days in setting A, a total of 141 showed a positive color reaction, most of which were classified as 1+ (n = 104, 73.8%). Reactions of 2+ and 3+ were seen in 24 (17.0%) and 13 (9.2%) of the tests, respectively. In setting B, 460 CCA-A tests were performed. Overall, 169 tests showed a positive reaction: 99 were judged 1+ (58.6%), 44 considered 2+ (26.0%) and the remaining 26 as 3+ (15.4%). Finally, in setting C, 76 (15.8%), 150 (31.1%) and 256 (53.1%) of the CCA-A tests performed were classified as 1+, 2+ and 3+, respectively.

The prevalence of *S. mansoni* according to a single CCA-B in setting A, B and C was 3.4%, 17.2% and 69.8%, respectively. In setting A, the five positive CCA-B tests were all judged 1+. In setting B, one CCA-B test result was considered 2+, whereas the remaining 21 test results were classified as 1+. Finally, in setting C, there were 116 CCA-B test results considered as 1+ (87.2%) and 17 as 2+ (12.8%).

#### Urine filtration and Hemastix results

In settings A, B and C, according to triplicate urine filtrations, the prevalence of *S. haematobium* was 4.1%, 0.8% and 65.3% in setting A, B and C, respectively. With regard to Hemastix dipstick results, after exclusion of trace results, the prevalence of microhematuria in settings A, B and C was 5.3%, 6.7% and 44.6%. Traces of microhematuria were additionally found in 33, 34 and 78 urines tested in setting A, B and C, respectively.

### Association between Kato-Katz and CCA Test Results

**Figure 2:**
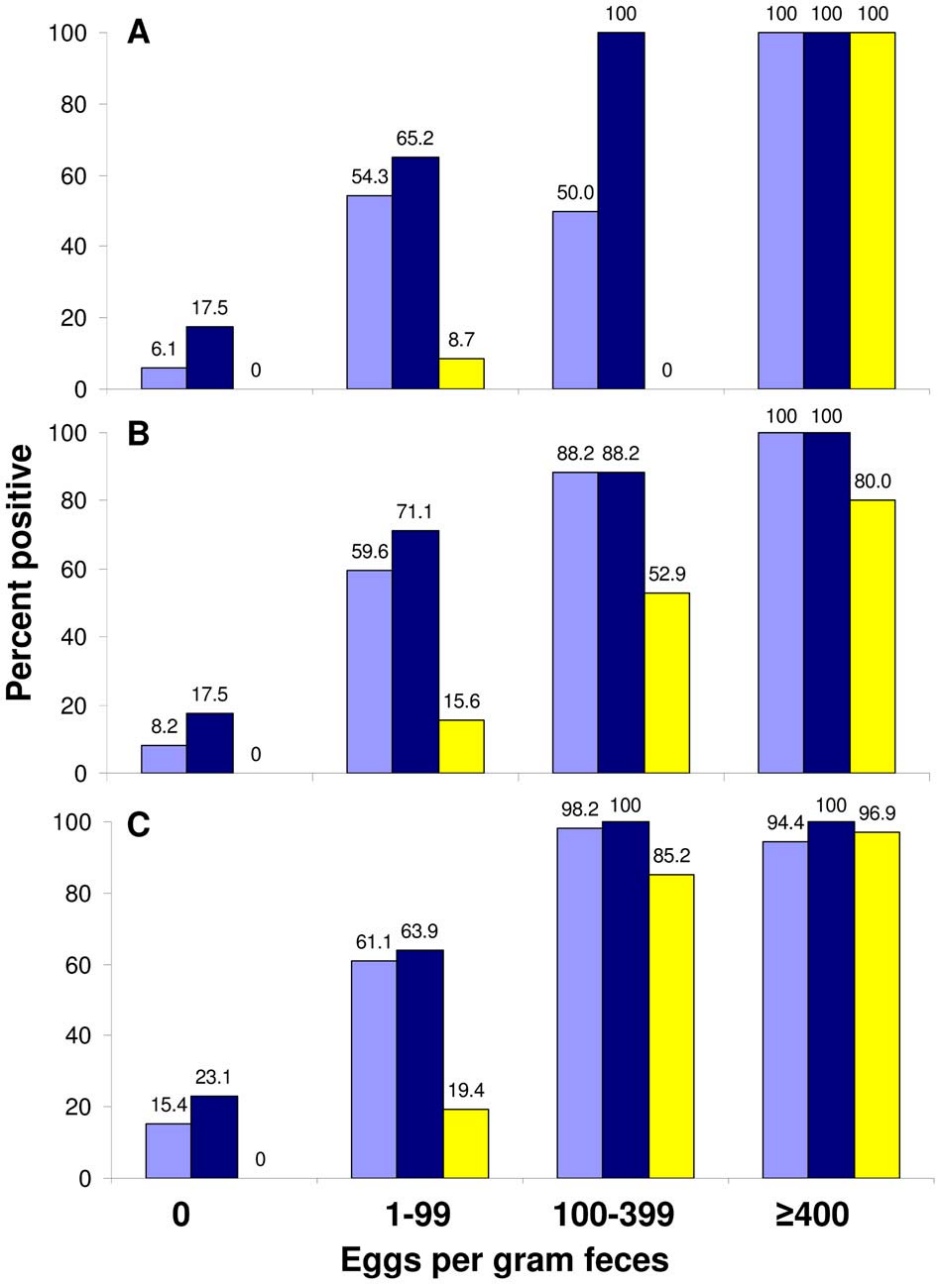
Correlation between Kato-Katz and CCA for *S. mansoni* diagnosis. Figure showing the correlation between the prevalence and intensity (stratified by intensity class) of *S. mansoni* infections, as determined by a single or triplicate CCA-A (light blue and dark blue bar, respectively), and a single CCA-B (yellow bar), stratified by study setting. According to nine Kato-Katz thick smear examinations, the prevalence of *S. mansoni* in setting A, B and C was 32.9%, 53.1% and 91.8%, respectively. In setting C, *S. haematobium* is co-endemic.

As indicated in Table 2, our ordinal logistic regression analysis showed that for an increase of *S. mansoni* infection intensity by 1 EPG, the likelihood of a stronger color reaction of the CCA-A (odds ratio (OR) = 1.07) and the CCA-B (OR = 1.03) is significant (both p<0.001). When *S. mansoni* FECs were not considered as continuous, but stratified according to pre-set thresholds into no, light, moderate and heavy infection intensity, we found that for each increase in infection intensity category, the likelihood of a stronger color reaction of both CCA-A (OR = 36.5) and CCA-B (OR = 25.2) is highly significant (both p<0.001). Figure 2 shows the correlation between infection intensity classes according to pre-set thresholds [22] and the percentage of infected individuals as determined by a single or triplicate CCA-A and a single CCA-B.

**Table 2:** Correlation of CCA test with schistosome infection and microhematuria (n = 526).

Test	Association	Adjusted OR (95% CI)	P-value
CCA-A			
(color categories)	*S. mansoni* egg count	1.07 (1.05, 1.09)	<0.001
	*S. mansoni* infection categories	36.50 (27.35, 48.77)	<0.001
CCA-A (pos/neg)	*S. haematobium* egg count	1.09 (0.97, 1.21)	0.121
	Hematuria trace	0.39 (0.09, 1.64)	0.200
	Hematuria moderate (2+)	1.09 (0.17, 7.00)	0.923
	Hematuria heavy (3+)	0.90 (0.12, 6.99)	0.195
CCA-B			
(color categories)	*S. mansoni* egg count	1.03 (1.01, 1.04)	<0.001
	*S. mansoni* infection categories	25.20 (15.83, 39.95)	<0.001
CCA-B (pos/neg)	*S. haematobium* egg count	NA*	
	Hematuria trace	NA*	
	Hematuria moderate (2+)	NA*	
	Hematuria heavy (3+)	NA*	

*NA: not applicable due to the small number of children without *S. mansoni* infection.

Ordinal logistic regression was used to assess the correlation between CCA test categories (0, 1+, 2+, and 3+) as outcome and *S. mansoni* egg count or *S. mansoni* infection categories (low, moderate, and heavy) as explanatory variable. Category “low” was used as baseline for comparison of other categories.

Logistic regression was applied to assess the correlation between CCA test results expressed as binary variables (positive/negative) and *S. haematobium* egg counts and microhematuria categories (trace, 1+, 2+, and 3+). Category “1+” was used as baseline for comparison of other categories.

### Diagnosis Accuracy of Different Tests

#### Agreement of the diagnostic assays

Table 3 shows the agreement between different diagnostic approaches and our first ‘gold’ standard (i.e., nine Kato-Katz thick smears derived from three stool samples) for the diagnosis of *S. mansoni*, stratified by study setting. The agreement between nine Kato-Katz thick smears and (i) a single Kato-Katz from the first stool sample was fair in setting A (κ = 0.31) and moderate in settings B and C (κ = 0.44–0.45), (ii) triplicate Kato-Katz from a single stool sample was moderate in settings A (κ = 0.55), and substantial in settings B and C (κ = 0.73), (iii) a single CCA-A was moderate in settings A, B and C (κ = 0.49–0.60), (iv) three CCA-A tests was moderate in settings A and C ( κ = 0.49–0.51) and substantial in setting B (κ = 0.61), (v) a single CCA-B was poor in setting A (κ = 0.14) and fair in settings B and C (κ = 0.26–0.33), and (vi) a single ether-concentration method was poor for all three settings (κ = 0.08–0.12).

**Table 3:** Agreement between different techniques for the diagnosis of *S. mansoni*.

Diagnostic approach	Test result	Setting A				Setting B				Setting C			
		Nine Kato-Katz thick smears				Nine Kato-Katz thick smears				Nine Kato-Katz thick smears			
		Positive	Negative	K*	P-values	Positive	Negative	K*	P-values	Positive	Negative	K*	P-values
One Kato-Katz thick smear	Positive	12	0			32	0			129	0		
	Negative	36	98	0.31	<0.001	37	61	0.45	<0.001	27	14	0.44	<0.001
Three Kato-Katz thick smears	Positive	23	0			51	0			147	0		
	Negative	25	98	0.55	<0.001	18	61	0.73	<0.001	9	14	0.73	<0.001
One CCA-A	Positive	27	6			48	5			138	2		
	Negative	21	92	0.54	<0.001	21	56	0.60	<0.001	16	11	0.49	<0.001
Three CCA-A	Positive	32	17			51	9			138	3		
	Negative	16	80	0.49	<0.001	15	48	0.61	<0.001	13	10	0.51	<0.001
One CCA-B	Positive	5	0			20	2			117	0		
	Negative	43	98	0.14	0.001	47	59	0.26	<0.001	39	14	0.33	<0.001
One ether-concentration	Positive	4	2			9	0			64	1		
	Negative	44	96	0.08	0.044	60	61	0.12	0.002	92	13	0.09	0.008

The study was carried out in three settings in south Côte d'Ivoire in October and November 2010. The κ-agreement of a single Kato-Katz thick smear from the first collected stool sample, triplicate Kato-Katz thick smears from the first collected stool sample, one CCA-A from the first collected urine sample, three CCA-A tests from three collected urine samples, one CCA-B test from the first collected urine sample, and one ether-concentration test on SAF-fixed stool samples from the second collected stool sample *versus* our diagnostic ‘gold’ standard of nine Kato-Katz thick smears (triplicate Kato-Katz thick smears from each of three stool samples) for the diagnosis of *S. mansoni* was calculated.

*Κ stands for kappa, κ<0 indicating no agreement, κ = 0–0.2 indicating poor agreement, κ = 0.21–0.4 indicating fair agreement, κ = 0.41–0.6 indicating moderate agreement, κ = 0.61–0.8 indicating substantial agreement, and κ = 0.81–1.0 indicating almost perfect agreement [27], [28].

#### Sensitivity and specificity of the diagnostic techniques

The sensitivity and specificity of the different diagnostic tests were determined for each of our two ‘gold’ standards (Table 4). In setting A, if the combined results of nine Kato-Katz thick smears were used as ‘gold’ standard, very low sensitivities of only 8.3% for a single ether-concentration test and 10.4% for a single CCA-B were determined. The highest sensitivity was revealed for triplicate CCA-A tests (66.7%), followed by duplicate (60.4%) and a single CCA-A test (56.3%).

**Table 4:** Sensitivity and specificity of different tests for the diagnosis of *S. mansoni*.

	Setting A		Setting B		Setting C	
Nine Kato-Katz thick smears as ‘gold’ standard	Sensitivity, % (CI*)	Specificity, % (CI*)	Sensitivity, % (CI*)	Specificity, % (CI*)	Sensitivity, % (CI*)	Specificity, % (CI*)
Single Kato-Katz	25.0 (13.6, 39.6)	100 (96.3, 100)	46.4 (34.3, 58.8)	100 (94.1, 100)	82.7 (75.8, 88.3)	100 (76.8, 100)
Duplicate Kato-Katz	29.2 (17.0, 44.1)	100 (96.3, 100)	55.1 (42.6, 67.1)	100 (94.1, 100)	86.5 (80.2, 91.5)	100 (76.8, 100)
Triplicate Kato-Katz	47.9 (33.3, 62.8)	100 (96.3, 100)	73.9 (61.9, 83.7)	100 (94.1, 100)	94.2 (89.3, 97.3)	100 (76.8, 100)
Single CCA-A	56.3 (41.2, 70.5)	93.9 (87.1, 97.7)	69.6 (57.3, 80.1)	91.8 (81.9, 97.3)	89.6 (83.5, 93.7)	84.6 (54.6, 98.6)
Duplicate CCA-A	60.4 (45.3, 74.2)	88.7 (80.6, 94.2)	77.6 (65.8, 86.9)	84.2 (72.1, 92.5)	91.5 (85.7, 95.3)	76.9 (46.2, 95.0)
Triplicate CCA-A	66.7 (51.6, 79.6)	82.5 (73.4, 89.4)	77.3 (65.3, 86.7)	84.2 (72.1, 92.5)	91.4 (85.7, 95.3)	76.9 (46.9, 95.0)
Single CCA-B	10.4 (3.4, 22.7)	100 (96.3, 100)	29.9 (19.3, 42.3)	96.7 (88.7, 100)	75.0 (67.4, 81.6)	100 (76.8, 100)
Single ether-concentration	8.3 (2.3, 20.0)	98.0 (92.9, 99.8)	13.0 (6.1, 23.3)	100 (94.1, 100)	41.0 (33.2, 49.2)	92.9 (66.1, 99.8)
**Combined results as ‘gold’ standard**						
Single Kato-Katz	17.9 (9.6, 29.2)	100 (95.4, 100)	40.0 (28.9, 52.0)	100 (92.3, 100)	80.5 (73.4, 86.5)	100 (69.2, 100)
Duplicate Kato-Katz	20.9 (11.9, 32.6)	100 (95.4, 100)	48.0 (36.3, 59.8)	100 (92.3, 100)	84.4 (77.7, 89.8)	100 (69.2, 100)
Triplicate Kato-Katz	34.3 (23.2, 46.9)	100 (95.4, 100)	64.0 (52.1, 74.8)	100 (92.3, 100)	92.2 (86.8, 95.9)	100 (69.2, 100)
Single CCA-A	47.8 (35.4, 60.3)	100 (95.4, 100)	65.3 (53.5, 76.0)	100 (92.3, 100)	89.0 (82.9, 93.4)	100 (69.2, 100)
Duplicate CCA-A	59.7 (47.0, 71.5)	100 (95.4, 100)	77.3 (66.2, 86.2)	100 (92.3, 100)	91.6 (86.0, 95.4)	100 (69.2, 100)
Triplicate CCA-A	73.1 (60.9, 83.2)	100 (95.4, 100)	77.3 (66.2, 86.2)	100 (92.3, 100)	91.6 (86.0, 95.4)	100 (69.2, 100)
Single CCA-B	7.5 (2.5, 16.6)	100 (95.4, 100)	28.0 (18.2, 39.6)	100 (92.3, 100)	72.7 (65.0, 79.6)	100 (69.2, 100)
Single ether-concentration	8.9 (3.4, 18.5)	100 (95.4, 100)	10.7 (4.7, 19.9)	100 (92.3, 100)	39.6 (31.8, 47.8)	100 (69.2, 100)

The study was carried out in three epidemiological settings of south Côte d'Ivoire in October and November 2010. Two different diagnostic ‘gold’ standards were applied to calculate sensitivity and specificity, namely (i) the combined results of nine Kato-Katz thick smears, and (ii) the combined results of all tests able to diagnose *S. mansoni* infections (i.e., nine Kato-Katz thick smears, one ether-concentration, three CCA-A, and one CCA-B).

*Exact 95% confidence interval.

In setting B, the lowest sensitivity of 13.0% was determined for a single ether-concentration test. The highest sensitivity was found for duplicate or triplicate CCA-A (both = 77%). Single CCA-A (69.6%), showed a slightly lower sensitivity than triplicate Kato-Katz thick smears (73.9%) from a single stool sample.

In setting C, the ether-concentration showed the lowest sensitivity (41.0%) for diagnosis of *S. mansoni*. A single (89.6%) and duplicate or triplicate CCA-A tests (both = 91%) did not show a higher sensitivity than triplicate Kato-Katz thick smears (94.2%).

Considering nine Kato-Katz thick smears as ‘gold’ standard, the specificity of triplicate CCA-A test was 82.5% (setting A), 84.2% (setting B), and 76.9% (setting C). The respective specificity of the CCA-B test was 100%, 96.7% and 100%.

Even when using a more rigorous diagnostic ‘gold’ standard (i.e., the combined results of nine Kato-Katz thick smears, plus triplicate CCA-A, a single CCA-B, and a single ether-concentration), very similar sensitivities were obtained.

### Effect of Concurrent *S. haematobium* Infection

Table 3 shows that, if only *S. mansoni*-negative children were included in a logistic regression analysis and adjustments were made for *S. haematobium* egg counts and infection intensity classes, no significant association between the CCA-A positivity rate and *S. haematobium* egg counts was found (OR = 1.09; p = 0.121). There was also no significant association between the CCA-A positivity rate and microhematuria classes detected (p>0.05). Due to the small number of children found positive with the CCA-B test no logistic regression analysis was performed.

## Discussion

For the rapid identification of populations at highest risk of schistosomiasis and other helminth infections that warrant preventive chemotherapy, as well as for monitoring progress of control interventions and new efforts toward elimination, assessment of drug efficacy, and improved patient management, the importance of an accurate diagnosis at the individual and population level must be emphasized [6], [7], [31]. The widely used Kato-Katz technique for the diagnosis of *S. mansoni* (and *S. japonicum*) has several shortcomings: in low endemicity settings this technique considerably underestimates the ‘true’ prevalence of infection [8], [11], [32]–[34]. Moreover, a minimum of equipment and well trained laboratory technicians are needed for quality results. Promising results have been reported with a CCA urine test for the diagnosis of *S. mansoni* in different settings [17], [35]. Some of the previous investigations, however, lacked a rigorous diagnostic ‘gold’ standard, as CCA test results were compared with singe or duplicate Kato-Katz thick smears from one or two stool samples [21], [36].

Within the frame of a SCORE-funded multi-country study, we have now assessed the accuracy of a commercially available CCA urine cassette assay (CCA-A, batch 32727) and an experimental formulation (CCA-B, batch 32686) provided by the same manufacturer and tuned to have a higher specificity, which was run in parallel with the commercially available test in three epidemiological settings of south Côte d'Ivoire. Results of the CCA tests were compared with nine Kato-Katz thick smears (three stool samples, each subjected to triplicate Kato-Katz thick smears). Additionally, we performed a single ether-concentration test using SAF-fixed stool samples. The influence of *S. haematobium* infection and presence of microhematuria on the performance of the CCA test was determined. In all three settings, a single CCA-A showed a similarly high sensitivity than triplicate Kato-Katz thick smears from a single stool sample, but both approaches missed a considerable number of infections when considering nine Kato-Katz thick smears as ‘gold’ standard. As expected, CCA-B showed a higher specificity than CCA-A, but the sensitivity of CCA-B was considerably lower than that of CCA-A. Indeed, a single CCA-B showed a significantly lower sensitivity than a single CCA-A, and triplicate Kato-Katz thick smears, particularly in settings A and B where the endemicity of *S. mansoni* was lower than in setting C. We were surprised by the low sensitivity of the ether-concentration method for *S. mansoni* diagnosis, which warrants follow-up investigations.

The CCA-A seems to be an appropriate test for the diagnosis of *S. mansoni* in our study area in south Côte d'Ivoire where the prevalence of *S. mansoni* is above 25% and no recent control efforts have been implemented. Importantly, the co-endemicity of *S. haematobium* did not influence the accuracy of the CCA-A for the diagnosis of *S. mansoni*. Additionally, a concurrent infection with soil-transmitted helminths showed no negative influence on the accuracy of the CCA urine test for *S. mansoni* diagnosis, confirming recent observations made by Shane and colleagues in a study done in Kenya [17]. Furthermore, our study did not reveal a significant association between CCA-A positive results and microhematuria, as determined by Hemastix dipsticks, which relaxes the manufacturer's indication that false-positive results can occur if an individual presents microhematuria. However, further studies in different settings are warranted to confirm that microhematuria or urinary tract infections are not negatively impacting on CCA test results. Also the ability of the CCA test to detect antigen of juvenile *Schistosoma* worms, which are not yet producing eggs, needs further investigation. Noteworthy, the sensitivity of 56.3% of a single CCA-A in the setting A with a *S. mansoni* prevalence of 32.9% (based on nine Kato-Katz thick smears) is considerably lower than the sensitivity of 96.3% detected with a single CCA cassette of the same manufacturer in a Kenyan setting with a similar prevalence (38.8%) [17]. This difference might be explained by our more rigorous diagnostic approach, i.e., triplicate instead of duplicate Kato-Katz thick smears of three consecutive stool samples as ‘gold’ standard and by working in a slightly lower endemicity area. The sensitivity of a single CCA-A for *S. mansoni* diagnosis increased from 56.3% (setting A) to 69.6% (setting B) and 89.6% (setting C) in parallel to increasing prevalence (32.9% to 53.1% and finally to 91.8%), and corresponding mean FECs (17.4 EPG to 62.4 EPG and finally to 482.8 EPG). These findings emphasize the impact of higher prevalences and infection intensities on the positivity rate of the CCA. The strong association between the intensity of the color reaction of the CCA-A band and *S. mansoni* infection intensities according to FECs by the Kato-Katz method in our studies is in line with previous reports of the CCA dipstick and cassette [37], [38]. The results of the experimental CCA-B formulation, which has been tested on a single urine sample from all children, are suboptimal. Indeed, only low sensitivities and a poor agreement with results of the Kato-Katz method were found, particularly in the lower endemicity areas (settings A and B). In our hands, despite high specificity, the CCA-B in its current formulation cannot be recommended for *S. mansoni* diagnosis in south Côte d'Ivoire.

The following issues speak for or against the application of the CCA-A *versus* the Kato-Katz method in helminth control programs or public health centers: at first view, in moderate-to-high-risk communities for *S. mansoni* infections as found in our study in Côte d'Ivoire (i.e., prevalence above 25%), the collection of a single stool sample and its examination with triplicate Kato-Katz thick smears seems to be an acceptable approach for *S. mansoni* diagnosis. The advantage of the Kato-Katz method is that it can concurrently detect other helminth species, such as the three main soil-transmitted helminths (i.e., *A. lumbricoides*, hookworm, and *T. trichiura*), which is not possible with the CCA. However, the Kato-Katz method requires a minimum of equipment, including a microscope, and well trained laboratory technicians who can identify helminth species-specific eggs in the thick smears. For application of the CCA-A, no additional equipment and only a minimum of training are needed. However, it only detects *S. mansoni* and no concurrent soil-transmitted helminth infections. The cost of a single cassette (approximately US$ 2) is currently still out of reach of people at highest risk of intestinal schistosomiasis (i.e., poor rural dwellers in sub-Saharan Africa) [17]. However, the cost of triplicate Kato-Katz thick smears are likely higher than a single CCA test [10]. From a convenience and logistical point of view, the collection of urine samples for the CCA is more straightforward than collection of stool for the Kato-Katz method. Indeed, urine production is more convenient for the patient and can be done without special efforts on the spot and at the same day resulting in high compliance rates, while stool production is inconvenient and collection can render a second consultation necessary and thus further exacerbate costs [8], [39].

The performance and sensitivity of the CCA test in low-risk communities (prevalence below 10%), identified by the application of multiple Kato-Katz thick smears on stool samples collected over multiple days, remains to be elucidated. Noteworthy, our study intended to test the CCA in a setting with a *S. mansoni* prevalence of 10–24% as requested by SCORE. However, we observed a considerable increase in the prevalence of *S. mansoni* when not only applying triplicate Kato-Katz from a single stool sample as in the pre-screening, but nine Kato-Katz thick smears overall from three stool samples: the observed prevalence increased from 17% to 34% in setting A, and from 36% to 54% in setting B. Due to this rigorous diagnostic approach we ended up with higher prevalences. Retrospectively, this had to be expected, as predicted by mathematical modeling and field observations [11], [13]. If the CCA test proves to be more sensitive than multiple Kato-Katz thick smears in settings characterized by low prevalence and intensity of *S. mansoni* infection intensities, it will be a most useful test. For example, in areas where intense helminth control efforts have diminished the prevalence and intensity of *S. mansoni* infections and control programs are focusing elimination, population screenings are necessary to identify remaining *S. mansoni* hot-spots for targeted anthelmintic treatment and other interventions. For these large-scale screenings the CCA-A would be an excellent tool due to its fast and easy application.

We conclude that in the current study area of south Côte d'Ivoire, where the prevalence and intensity of *S. mansoni* are still high, partially explained by the prior lack of control efforts, the CCA-A can become a useful method for *S. mansoni* diagnosis in health centers at the periphery and schistosomiasis control programs. On the other hand, while the specificity of the CCA-B test was high, its current formulation cannot be recommended for *S. mansoni* diagnosis. Clearly, there is a need to evaluate the CCA test in settings characterized by low *S. mansoni* prevalences and infection intensities to assess its potential role in schistosomiasis control programs progressing toward transmission control and local elimination and for reliable individual diagnosis.

## Supporting Information

Alternative Language Abstract S1 **Genauigkeit des im Urin zirkulierenden kathodischen Antigen (CCA) Tests für die Diagnose von** ***Schistosoma mansoni*** **Infektionen in verschiedenen Gebieten der Côte d'Ivoire - Translation of abstract into German by Stefanie Knopp.**
(DOC)

Alternative Language Abstract S2 **Précision d'un test basé sur la détection d'antigènes cathodiques circulants (ACC) dans l'urine pour le diagnostic de** ***Schistosoma mansoni*** **dans différents foyers en Côte d'Ivoire - Translation of abstract into French by Jean T. Coulibaly and Kigbafori D. Silué.**
(DOC)

Checklist S1 **STARD checklist.**
(DOC)
