# Supplementary material for: Accuracy of Urine Circulating Cathodic Antigen (CCA) Test for Schistosoma mansoni Diagnosis in Different Settings of Côte d'Ivoire
Source: PLoS Negl Trop Dis. 2011 Nov 22;5(11):e1384. doi: 10.1371/journal.pntd.0001384 (PMC3222626; doi:10.1371/journal.pntd.0001384)
Supplement: Alternative Language Abstract S1 — Genauigkeit des im Urin zirkulierenden kathodischen Antigen (CCA) Tests für die Diagnose von Schistosoma mansoni Infektionen in verschiedenen Gebieten der Côte d'Ivoire - Translation of abstract into German by Stefanie Knopp. (DOC) [file pntd.0001384.s001.doc]

**Précision d’un test basé sur la détection d’antigènes cathodiques circulants (ACC) dans l’urine pour le diagnostic de *Schistosoma mansoni* dans différents foyers en Côte d’Ivoire**

***Contexte:*** Des résultats prometteurs ont été rapportés pour un test de diagnostic de *Schistosoma mansoni* basé sur la détection des antigènes cathodiques circulants (ACC). Nous avons évalué la précision d’un test de diagnostic rapide disponible (désigné ACC-A) et un autre test au stade expérimental (ACC-B) utilisés tous deux pour le diagnostic de *S*. *mansoni.*

***Méthodologie:*** Nous avons conduit une étude transversale dans trois zones épidémiologiques en Côte d’Ivoire : les zones A et B sont endémiques pour *S. mansoni*, tandis que *S. haematobium* est co-endémique dans la zone C. Au total, 446 élèves âgés de 8-12 ans ont fourni trois échantillons de selles et d’urine. Trois lames de Kato-Katz par échantillon de selles ont été examinées pour le diagnostic de *S. mansoni*, tandis que les échantillons d’urine ont été testés avec le test rapide (ACC-A). En plus, les échantillons de selles et d’urine du premier jour ont été respectivement soumis à la technique d’éther-concentration et au test expérimental de ACC-B. Les échantillons d’urine ont été examinés par la méthode de filtration pour le diagnostic de *S. haematobium* et par les bandelettes réactives pour la micro-hématurie.

***Principales Résultats:*** Considérant les neuf lames de Kato-Katz comme référence, la prévalence de *S. mansoni* dans les zones A, B et C était 32,9 % ; 53,1 % et 91,8 % respectivement. La sensibilité des trois lames de Kato-Katz de l’échantillon de selles du premier jour et un test ACC-A était 47,9 % et 56,3 % dans la zone A ; 73,9 % et 69,6 % dans la zone B et 94,2 % et 89,6 % dans la zone C. La sensibilité d’un test ACC-B était 10,4 % ; 29,9 % et 75,0 % respectivement. La sensibilité de la technique d’éther-concentration pour le diagnostic de *S. mansoni* était faible (8,3-41,0 %). La spécificité du test ACC-A était modérée (76,9-84,2 %), tandis qu’une haute spécificité a été obtenue pour le test ACC-B (96,7-100 %). La probabilité de l’intensité de la couleur des bandes du test ACC-A augmente avec le nombre d’œufs de *S. mansoni* (odds ratio : 1,07 ; p <0.001). Une co-infestation avec *S. haematobium* ou la présence de micro-hématurie n’influence pas les résultats du test ACC-A pour le diagnostic de *S. mansoni*.

***Conclusion:*** Le test ACC-A a montré une sensibilité similaire à celle de trois lames de Kato-Katz pour le diagnostic de *S. mansoni*. Il n’y a pas de réaction croisée avec *S. haematobium* et la micro-hématurie. La faible sensibilité du test ACC-B dans notre zone d’étude ne préconise pas son usage pour le diagnostic de *S. mansoni*.

***Traduction:*** Jean T. Coulibaly et Kigbafori D. Silué
