# Supplementary material for: Accuracy of Urine Circulating Cathodic Antigen (CCA) Test for Schistosoma mansoni Diagnosis in Different Settings of Côte d'Ivoire
Source: PLoS Negl Trop Dis. 2011 Nov 22;5(11):e1384. doi: 10.1371/journal.pntd.0001384 (PMC3222626; doi:10.1371/journal.pntd.0001384)
Supplement: Alternative Language Abstract S2 — Précision d'un test basé sur la détection d'antigènes cathodiques circulants (ACC) dans l'urine pour le diagnostic de Schistosoma mansoni dans différents foyers en Côte d'Ivoire - Translation of abstract into French by Jean T. Coulibaly and Kigbafori D. Silué. (DOC) [file pntd.0001384.s002.doc]

**Genauigkeit des im Urin zirkulierenden kathodischen Antigen (CCA) Tests für die Diagnose von *Schistosoma mansoni* Infektionen in verschiedenen Gebieten der Côte d’Ivoire**

**Zusammenfassung**

***Hintergrund:*** Viel versprechende Ergebnisse wurden für einen zirkulierenden kathodischen Antigen (CCA) Test zur Diagnose von *Schistosoma mansoni* veröffentlicht. Wir haben nun die Genauigkeit eines kommerziell erhältlichen Kassetten Tests (bezeichnet als CCA-A) und einer experimentellen Formulierung (CCA-B) zur Diagnose von *S.* *mansoni* untersucht.

***Methodologie:*** Wir führten eine Querschnittsstudie in drei epidemiologisch unterschiedlichen Gebieten der Côte d’Ivoire durch: Gebiet A und B sind endemisch für *S. mansoni*, wohingegen *S. haematobium* in Gebiet C ebenfalls vorkommt. Insgesamt übergaben uns 446 Kinder im Alter von 8-12 Jahren mehrere Stuhl- und Urinproben. Zur Diagnose von *S. mansoni* wurden die Stuhlproben mit jeweils drei Kato-Katz Ausstrichen und die Urinproben mit dem CCA-A untersucht. Die erste Stuhl- und Urinprobe wurden zusätzlich mit einer Ether-Konzentrations-Methode und dem CCA-B getestet. Zur Diagnose von *S. haematobium* wendeten wir eine Urinfiltrationsmethode an. Zur Messung von Mikrohämaturie wurde ein Hemastix Schnelltest benutzt.

***Wichtigste Ergebnisse:*** Neun Kato-Katz Ausstriche wurden als diagnostischer Goldstandard betrachtet. Die *S. mansoni* Prävalenz in Gebiet A, B und C war 32.9%, 53.1% und 91.8%. Die Untersuchung der ersten Stuhlprobe ergab für dreifache Kato-Katz Ausstriche und einen einzelnen CCA-A Test eine Sensitivität von 47.9% und 56.3% (Gebiet A), 73.9% und 69.6% (Gebiet B), und 94.2% and 89.6% (Gebiet C). Die Sensitivität für einen CCA-B war 10.4%, 29.9% und 75.0% in den jeweiligen Gebieten. Die Ether-Konzentrationsmethode resultierte in einer niedrigen Sensitivität (8.3-41.0%) für die Diagnose von *S. mansoni*. Die Spezifität des CCA-A war mittelprächtig (76.9-84.2%), die des CCA-B war hoch (96.7-100%). Die Wahrscheinlichkeit einer CCA-A Farbreaktion wurde mit ansteigenden *S. mansoni* Eizahlen höher (Odds Ratio: 1.07, p<0.001). Eine gleichzeitige Infektion mit *S. haematobium* oder das Vorhandensein von Mikrohämaturie hatte keinen Einfluss auf die CCA-A Testergebnisse für *S. mansoni*.

***Schlussfolgerung/Bedeutung:*** Die Sensitivität des CCA-A Tests zur Diagnose von *S. mansoni* Infektionen war mit der eines dreifachen Kato-Katz Ausstriches vergleichbar. Der CCA-A Test zeigte keine Kreuzreaktion mit *S. haematobium* Infektionen und Mikrohämaturie. Die niedrige Sensitivität des CCA-B in unserem Studiengebiet schliesst seine Anwendung zur Diagnose von *S. mansoni* aus.

***Übersetzung:*** Stefanie Knopp
